# The expression and prognostic value of the epidermal growth factor receptor family in glioma

**DOI:** 10.1186/s12885-021-08150-7

**Published:** 2021-04-23

**Authors:** Bin Xu, Zhengyuan Huo, Hui Huang, Wei Ji, Zheng Bian, Jiantong Jiao, Jun Sun, Junfei Shao

**Affiliations:** Department of Neurosurgery, Wuxi People’s Hospital Affiliated to Nanjing Medical University, No. 299 Qing Yang Road, Wuxi, 214023 Jiangsu China

**Keywords:** Glioma, EGFR family, Prognosis, Immune infiltration

## Abstract

**Background:**

The epidermal growth factor receptor (EGFR) family belongs to the transmembrane protein receptor of the tyrosine kinase I subfamily and has 4 members: EGFR/ERBB1, ERBB2, ERBB3, and ERBB4. The EGFR family is closely related to the occurrence and development of a variety of cancers.

**Materials/methods:**

In this study, we used multiple online bioinformatics websites, including ONCOMINE, TCGA, CGGA, TIMER, cBioPortal, GeneMANIA and DAVID, to study the expression profiles, prognostic values and immune infiltration correlations of the EGFR family in glioma.

**Results:**

We found that EGFR and ERBB2 mRNA expression levels were higher in glioblastoma (GBM, WHO IV) than in other grades (WHO grade II & III), while the ERBB3 and ERBB4 mRNA expression levels were the opposite. EGFR and ERBB2 were notably downregulated in IDH mutant gliomas, while ERBB3 and ERBB4 were upregulated, which was associated with a poor prognosis. In addition, correlation analysis between EGFR family expression levels and immune infiltrating levels in glioma showed that EGFR family expression and immune infiltrating levels were significantly correlated. The PPI network of the EGFR family in glioma and enrichment analysis showed that the EGFR family and its interactors mainly participated in the regulation of cell motility, involving integrin receptors and Rho family GTPases.

**Conclusions:**

In summary, the results of this study indicate that the EGFR family members may become potential therapeutic targets and new prognostic markers for glioma.

## Background

Brain and central nervous system (CNS) cancers (collectively referred to as CNS cancers) were responsible for substantial morbidity and mortality worldwide between 1990 and 2016 [1]. Glioma is a common brain tumor in humans and is one of the most malignant tumors among all cancers [2, 3]. Although various treatments for glioma, including surgery, radiotherapy, systemic therapy, tumor treatment fields, and supportive treatment, have recently made progress, the median survival period after diagnosis is still approximately 15 months, and long-term survival is unsatisfactory [4]. Because current treatments cannot significantly improve patient outcomes, the discovery of novel treatment strategies is critical. Therefore, the identification of new biomarkers is of great significance for improving the prognosis and developing individualized treatment.

The epidermal growth factor receptor (EGFR) family (also known as the HER family) belongs to the transmembrane protein receptor of the tyrosine kinase I subfamily, and it has 4 members: EGFR/ERBB1, ERBB2, ERBB3, and ERBB4, encoded by the proto-oncogenes ERBB1–4. The members of the EGFR family are similar in structure and consist of an extracellular ligand-binding domain, a hydrophobic transmembrane region and an intracellular segment containing a conserved tyrosine kinase domain [5, 6]. After the ligand binds to the extracellular domain, the protein conformation of the extracellular domain is changed. Phosphorylation of the tyrosine kinase in the intracellular domain initiates the signal transduction pathway, transmits the signal from outside the cell into the cytoplasm, and modulates the cell’s response to external stimuli, thereby regulating the growth, survival, transformation and apoptosis of normal cells [7, 8].

Overexpression and activation of the EGFR family can be seen in many human cancers and they are closely related to the clinicopathological characteristics and prognosis of many tumors, such as breast cancer [9], lung cancer [10], gastric cancer [11] and melanoma [12]. Previous studies have discovered EGFR family pathway dysregulation in gliomas and their relationship with the clinical characteristics and prognosis of human gliomas. However, the expression patterns and roles of the EGFR family proteins in gliomas are issues that urgently need attention.

In recent years, due to the continuous development and application of bioinformatics databases, an increasing number of tumor biomarkers have been discovered [13–16]. In addition, an increasing number of studies have shown that the EGFR family can be used as potential targets for the treatment of glioma [17, 18]. In this study, we downloaded EGFR family expression data from various online databases and analyzed the relationship between their transcription levels in gliomas and the clinical prognosis. Analysis of the tumor immune estimation resource (TIMER) database revealed a correlation between the EGFR family and tumor infiltrating immune cells in the tumor microenvironment. Our research shows that the EGFR family members may be potential therapeutic targets with promising prognostic value in glioma patients.

## Materials and methods

### Oncomine database analysis

We used the Oncomine database (https://www.oncomine.org/) [19] to extract the data of the expression levels of the EGFR family in various types of glioma tissues. Then, we analyzed the differential expression of the members of the EGFR family between cancer tissue and normal tissue through Student’s t-test. Critical value setting conditions: Fold change> 1.5, *P*-value< 0.01.

### Acquisition of the data from the TCGA and CGGA dataset

The RNA sequencing data and clinical information in the TCGA-GBMLGG dataset were downloaded from UCSC Xena (https://xenabrowser.net/datapages/) [20]. In addition, the RNA sequencing data and clinical information in the CGGA dataset (mRNAseq_325) were also obtained from their official website (http://www.cgga.org.cn/index.jsp) [21]. For further analysis, a total of 668 samples from the TCGA dataset and 326 primary glioma samples from the CGGA dataset, which contained both gene expression and survival data, were extracted.

### Tumor infiltrating immune cells analysis

The TIMER database (https://cistrome.shinyapps.io/timer/) is a database that can comprehensively and systematically analyze the interaction between tumors and immunity [22]. We downloaded the estimated data of tumor-infiltrating immune cells from the TIMER database and analyzed the correlation between the expression levels of the EGFR family members and the abundance of infiltrating immune cells in glioma.

### cBioPortal analysis

cBioPortal (https://www.cbioportal.org/) provides a visual tool for research and analysis of cancer gene data and helps cancer tissue and cytology research gain molecular data understanding of their genetics, epigenetics, gene expression and proteomics. We can study the link between genetic changes and clinical practice by customizing the interface of the data. Through the cBioPortal online tool, we analyzed EGFR family alterations and their correlations with clinical factors. We used the glioma dataset for analysis of EGFR family expression with cBioPortal [23].

### GeneMANIA analysis

The GeneMANIA database (http://www.genemania.org/) is a website dedicated to the study of protein-protein interaction (PPI) relationships [24]. It mainly provides data predictions including the following: protein predictions, protein interactions, coexpression, sharing of protein domains, subcellular colocalization, signaling pathways, genetic interactions, etc. and it can construct a PPI network. In this study, humans (*Homo sapiens*) were selected in the species selection interface to search for proteins interacting with members of the EGFR family.

### DAVID analysis

DAVID (https://david.ncifcrf.gov/) is a public database that integrates biological data and analysis tools and can annotate genes and pathways [25]. GO is a bioinformatics tool that annotates genes and analyzes the biological processes in which they participate. KEGG is a database used to analyze the relevant signaling pathways in a large-scale molecular data set generated by high-throughput experimental technology. DAVID was used for GO enrichment analysis of the EGFR family in three aspects: molecular function (MF), cell composition (CC) and biological process (BP), as well as the enrichment analysis of KEGG pathways, to clarify the gene function and the cell signaling pathways of the members of the EGFR family.

### Statistical analysis

Student’s t-test was used to analyze gene expression in the Oncomine, TCGA and CCGA databases as well as IDH wild-type and mutation data in the TCGA and CCGA databases. The survival curves were compared using the log-rank test. Spearman’s correlation analysis was used in the TIMER database. *P* < 0.05 was considered statistically significant.

## Results

### The mRNA expression levels of the EGFR family across different types of cancers

**Fig. 1:**
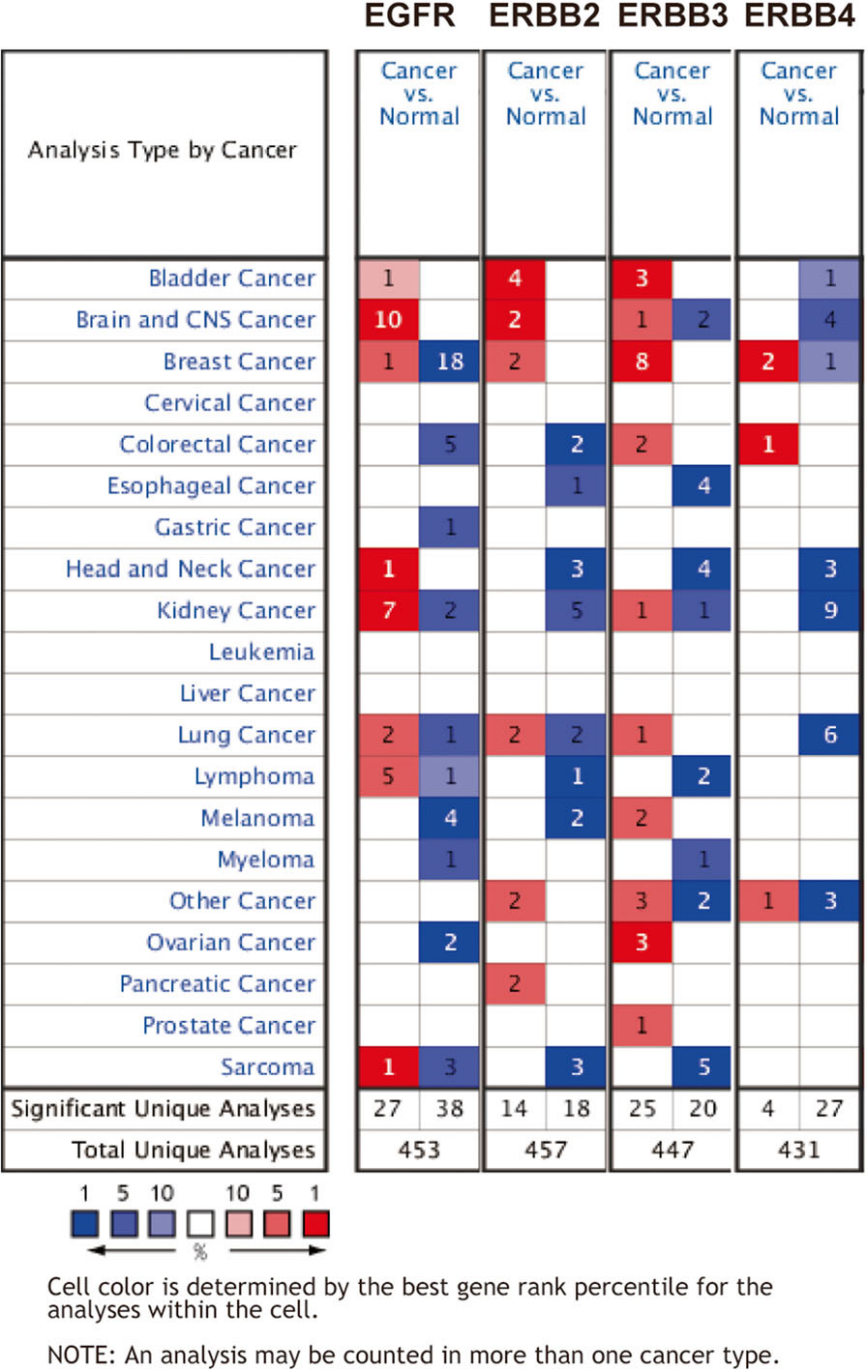
The expression of the EGFR family in different cancers

The Oncomine database was used to compare the mRNA expression levels of the EGFR family between tumor and normal tissues. This analysis revealed that EGFR family expression was significantly different in glioma tissues compared with normal tissues (Fig. 1b). According to the information from the datasets in Oncomine, in Sun’s datasets [26], the mRNA levels of EGFR were 9.390, 5.740, and 8.211 times higher in glioma tissues with different histological types than in normal tissues (Table 1). In the TCGA dataset, the expression of EGFR was 3.792- and 2.956-fold higher in glioma tissues with different histological types than in normal tissues (Table 1). In French’s dataset [27], the expression of EGFR was 9.847 times higher in anaplastic oligodendroglioma tissues than in normal tissues (Table 1). In Lee’s dataset [28], the expression of EGFR was 3.772 times higher in glioblastoma tissues than in normal tissues (Table 1). In Shai’s dataset [29], the expression of EGFR was 3.815 times higher in glioblastoma tissues than in normal tissues (Table 1). In Bredel’s dataset [30], the expression of EGFR was 5.840 times higher in glioblastoma tissues than in normal tissues (Table 1). In Murat’s dataset [31], the expression of EGFR was 10.667 times higher in glioblastoma tissues than in normal tissues (Table 1). In Watson’s dataset [32], the expression of ERBB2 was 5.166 times higher in meningioma tissues than in normal tissues (Table 1). In Bredel’s dataset [33], the expression of ERBB2 was 3.065 times higher in glioblastoma tissues than in normal tissues (Table 1). In Pomeroy’s dataset [34], the expression of ERBB3 was 8.973 times higher in classic medulloblastoma tissues than in normal tissues (Table 1). ERBB4 had no available research results that met the screening criteria.

**Table 1:** In different types of gliomas and normal brain tissues, differences in the transcriptional levels of the EGFR family

EGFR family	Type of glioma vs. brain	Fold change	P	t-test	Reference
EGFR	Glioblastoma vs. Normal	9.390	3.09E-27	14.885	Sun [14]
	Oligodendroglioma vs. Normal	5.740	1.39E-15	10.540	Sun [14]
	Anaplastic Astrocytoma vs. Normal	8.211	9.39E-8	7.824	Sun [14]
	Brain Glioblastoma vs. Normal	3.792	5.10E-18	14.875	TCGA
	Anaplastic Oligodendroglioma vs. Normal	9.847	4.07E-9	9.400	French [15]
	Glioblastoma vs. Normal	3.772	9.78E-7	8.111	Lee [16]
	Glioblastoma vs. Normal	3.815	2.75E-5	4.729	Shai [17]
	Glioblastoma vs. Normal	5.840	9.51E-7	6.098	Bredel [18]
	Brain Glioblastoma vs. Normal	2.956	9.32E-101	26.116	TCGA
	Glioblastoma vs. Normal	10.667	1.16E-6	10.112	Murat [19]
ERBB2	Meningioma vs. Normal	5.166	8.89E-7	7.324	Watson [20]
	Glioblastoma vs. Normal	3.065	1.70E-9	10.222	Bredel [18]
ERBB3	Classic Medulloblastoma vs. Normal	8.973	3.79E-8	6.404	Pomeroy [21]
ERBB4	NA	NA	NA	NA	NA

### Subtype analysis of mRNA expression levels of the EGFR family in glioma

**Fig. 2:**
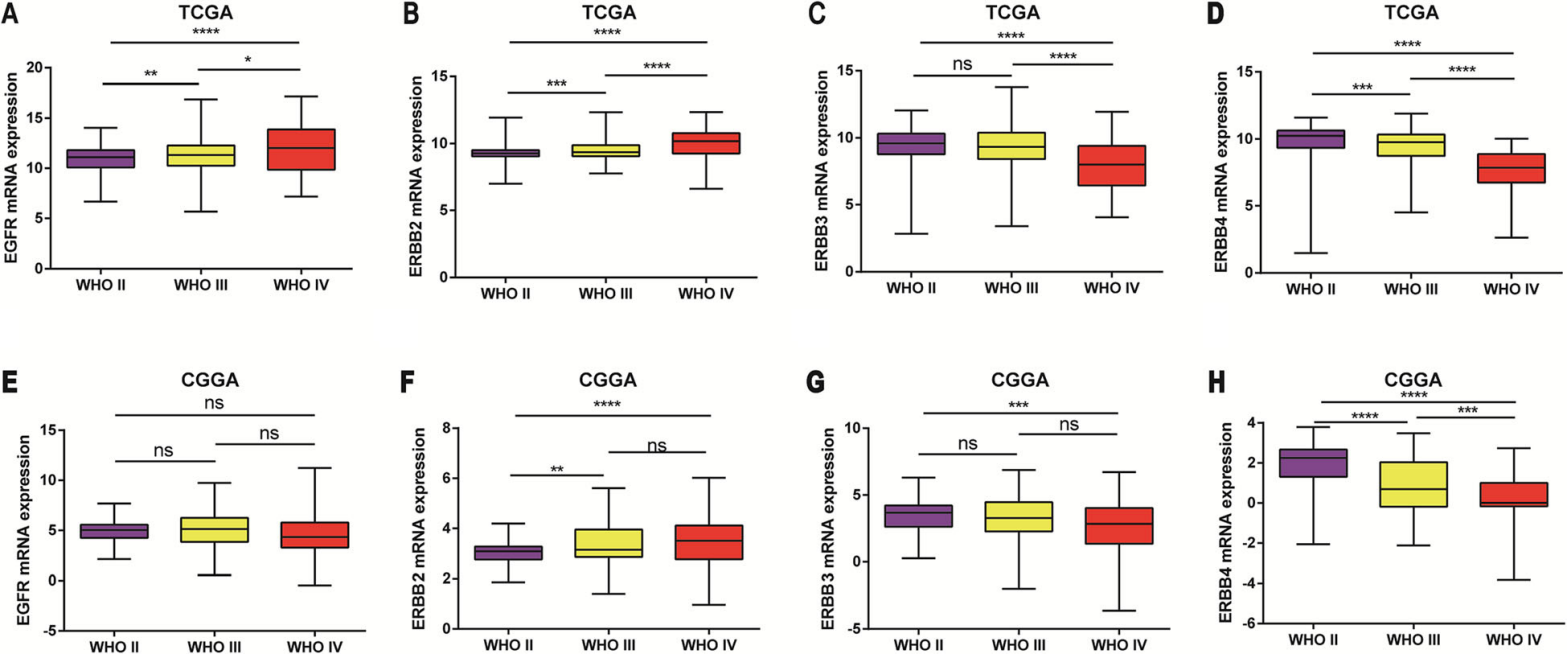
The expression level of the EGFR family in different grades of glioma tissues. **a, b, e** and **f** The mRNA expression levels of EGFR and ERBB2 were significantly increased in GBM. **c, d, g**, and **h** The mRNA expression levels of ERBB3 and ERBB4 were significantly increased in glioma (WHO II). * represents *p* < 0.05, ** represents *p* < 0.01, *** represents *p* < 0.001, and **** represents *p* < 0.0001

To analyze the transcription levels of the EGFR family in subtypes of glioma patients, the TCGA and CGGA databases were applied. According to the tumor grades, in the TCGA database, compared with WHO II & III, the EGFR transcription level was the highest in WHO IV (Fig. 2a). However, by analyzing the CGGA RNA-seq database, we found that this difference was not statistically significant (Fig. 2e). In the TCGA database, the transcription level of ERBB2 was the highest in WHO IV compared with WHO II & III (Fig. 2b). However, the transcription levels of ERBB3 and ERBB4 in WHO IV were significantly lower than those in II & III and this difference was statistically significant (Fig. 2c and d). Analysis of the CGGA RNA-seq data set also found that the transcription levels of ERBB2, ERBB3 and ERBB4 were similar (Fig. 2f, g and h). In summary, the mRNA levels of EGFR and ERBB2 were higher in advanced and poorly differentiated gliomas; however, the mRNA levels of ERBB3 and ERBB4 were lower in advanced and poorly differentiated gliomas.

**Fig. 3:**
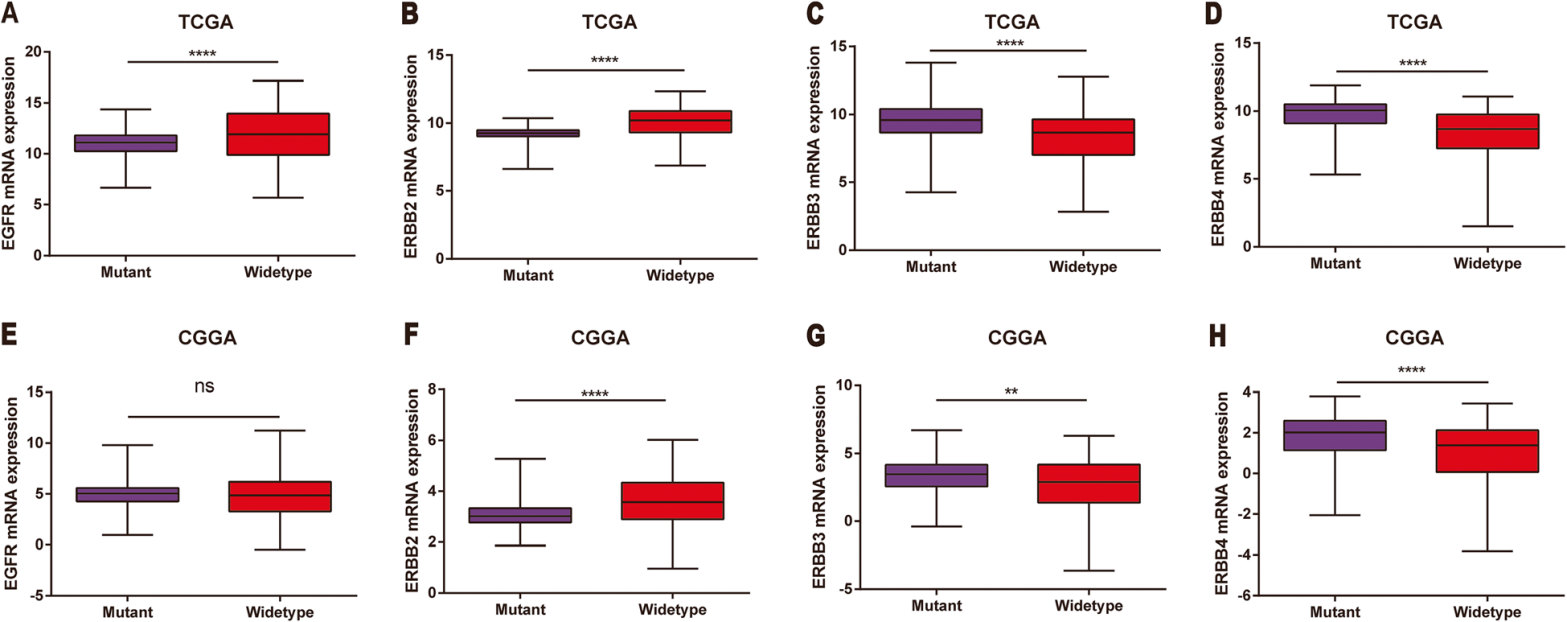
EGFR family expression was closely associated with IDH wild-type and mutation in glioma. **a**, **b**, **e** and **f** EGFR and ERBB2 were notably downregulated in IDH mutant glioma. **c**, **d**, **g** and **h** ERBB3 and ERBB4 were notably upregulated in IDH mutant glioma. * represents *p* < 0.05, ** represents *p* < 0.01, *** represents *p* < 0.001, and **** represents *p* < 0.0001

**Fig. 4:**
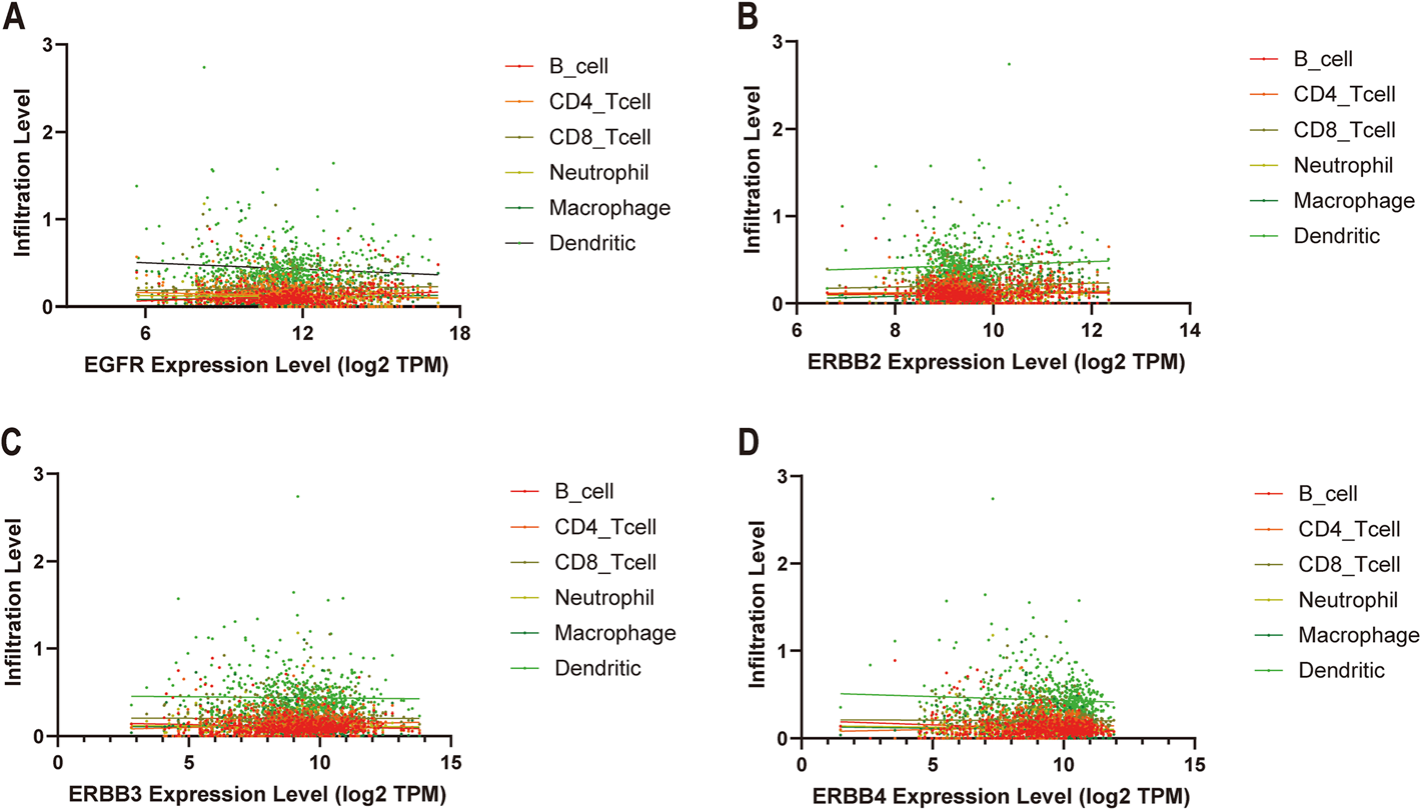
Correlation between the transcription level of the EGFR family and the immune infiltration level in glioma. **a** The transcription level of EGFR was positively correlated with the level of B cell infiltration in tumor tissues. **b** The transcription level of ERBB2 EGFR was positively correlated with the level of macrophage infiltration in tumor tissues. **c** and **d** The transcriptional expression levels of ERBB3 and ERBB4 were positively correlated with the level of CD4+ T cell infiltration in tumor tissues

Mutations in isocitrate dehydrogenase (IDH) play an important role in the occurrence and development of glioma and serve as a potential prognostic marker for patients with glioma [35]. Therefore, we studied the expression level of EGFR in IDH mutant and wild type. In the TCGA data, the expression level of EGFR in IDH wild-type glioma was elevated (Fig. 3). However, in the CGGA RNA-seq data set, there was no significant difference in the expression level of EGFR in IDH wild-type glioma (Fig. 4a, e). In the TCGA data, the expression level of ERBB2 in IDH wild-type gliomas was notably increased (Fig. 1, 4b), and it was also increased in the CGGA RNA-seq datasets (Fig. 4f). Analysis of the TCGA data and the CGGA RNA-seq data found that ERBB3 and ERBB4 were notably increased in IDH mutant gliomas (Fig. 1, 4b, c and d), and the same result was found in the CGGA RNA-seq datasets (Fig. 4g and h). In conclusion, data analysis shows that the expression levels of the members of the EGFR family are notably different in different IDH states and they have the potential for use as biomarkers of IDH subtypes of glioma.

### Correlation between EGFR family expression and immune infiltrating levels in glioma

**Fig. 5:**
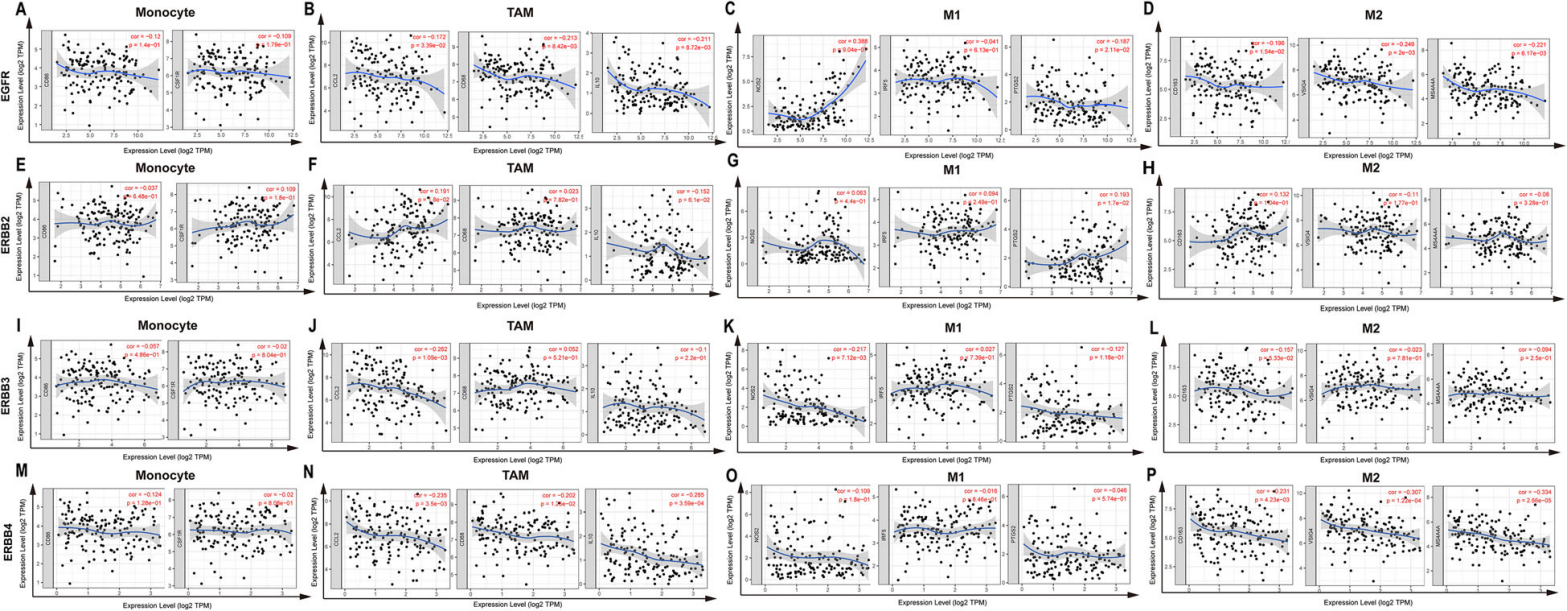
EGFR family expression was correlated with macrophage polarization in glioma. Markers include CD86 and CSF1R of monocytes; CCL2, CD68, and IL10 of TAMs (tumor-associated macrophages); NOS2, IRF5, and PTGS2 of M1 macrophages; and CD163, VSIG4, and MS4A4A of M2 macrophages in glioma (*n* = 153). **a**-**d** Scatterplots of correlations between EGFR expression and gene markers of monocytes, TAMs, and M1 and M2 macrophages. **e**-**h** Scatterplots of correlations between ERBB2 expression and gene markers of monocytes, TAMs, and M1 and M2 macrophages. **i**-**l** Scatterplots of correlations between ERBB3 expression and gene markers of monocytes, TAMs, and M1 and M2 macrophages. **m**-**p** Scatterplots of correlations between ERBB4 expression and gene markers of monocytes, TAMs, and M1 and M2 macrophages

**Fig. 6:**
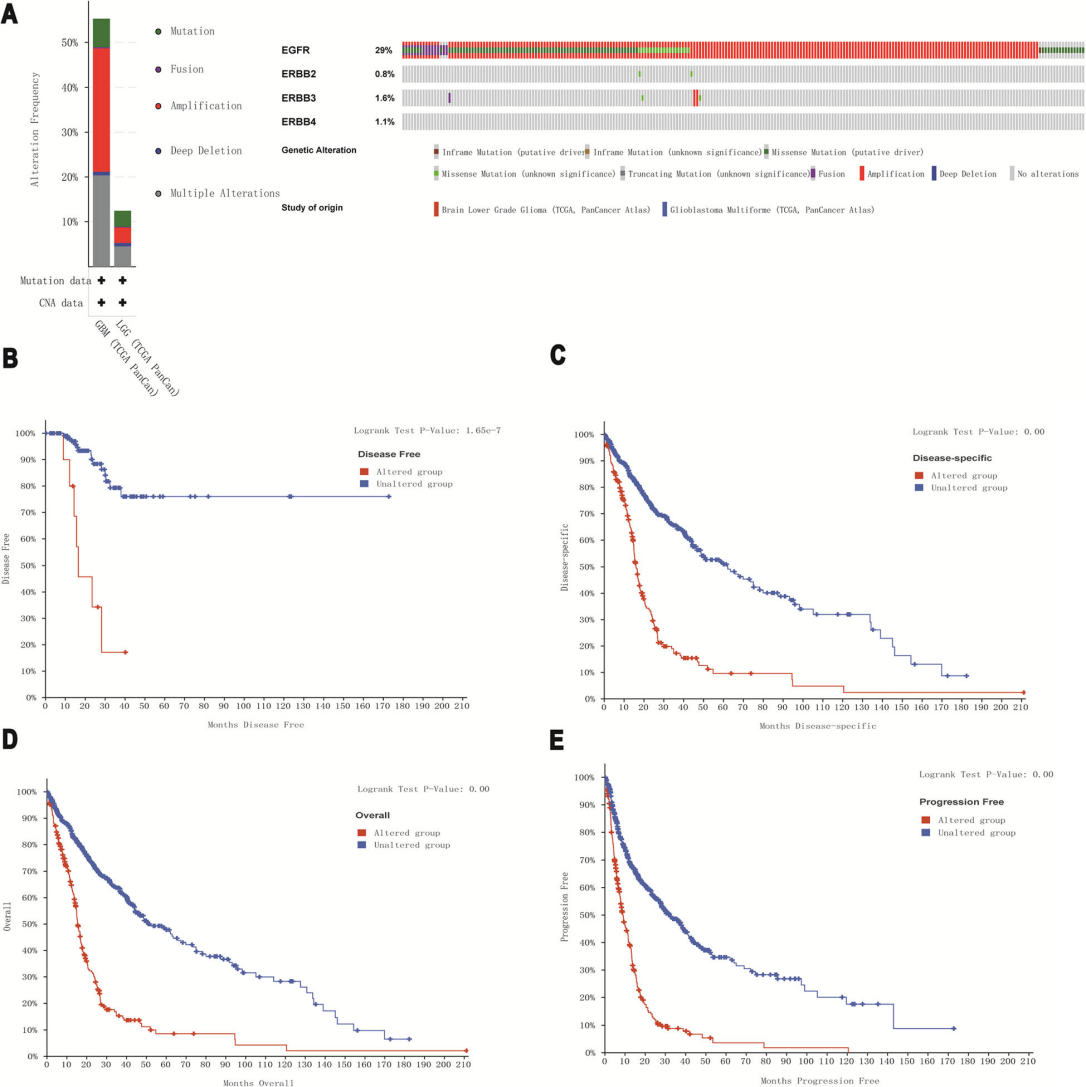
Relationship between EGFR family mutations and prognosis in gliomas. **a** The mutation rates of EGFR, ERBB2, ERBB3 and ERBB4 were 29, 0.8, 1.6 and 1.1%, respectively. **b**, **c**, **d** and **e** EGFR family mutations in glioma patients predicted poor OS, DFS, DS, and PFS

An increasing number of studies have shown that tumor-infiltrating lymphocytes can be used as related indicators to predict tumor metastasis and invasion [36, 37]. Therefore, by analyzing the TIMER database, we found a correlation between the expression levels of the members of the EGFR family and the level of immune infiltration in glioma tissues. As shown in Fig. 5 and Table 2, the expression level of EGFR mRNA was notably positively correlated with the level of B cell infiltration in glioma tissue (r = 0.1671, *p* < 0.0001). The expression level of EGFR mRNA was notably negatively correlated with the infiltration level of DCs (r = − 0.09997, *p* = 0.0088) and CD4+ T cells (r = − 0.1143, *p* = 0.0027) in glioma tissue. ERBB2 mRNA expression was notably positively correlated with the level of macrophage infiltration in gliomas (r = 0.1026, *p* = 0.0072). The mRNA expression of ERBB3 and ERBB4 in gliomas was notably positively correlated with the level of CD4+ T cell infiltration (ERBB3, r = 0.1200, *p* = 0.0016, ERBB4, r = 0.09663, *p* = 0.0114). The expression of ERBB3 and ERBB4 mRNA in gliomas was notably negatively correlated with the level of B cell infiltration (ERBB3, r = − 0.08882, *p* < 0.0201, ERBB4, r = − 0.1591, *p* < 0.001). However, the glioma control group showed that ERBB2 expression had no significant correlation with macrophage polarization in gliomas (Fig. 6a-o). These results strongly suggest that the members of the EGFR family play specific roles in regulating the immune infiltration of glioma.

**Table 2:** Correlation between EGFR family mRNA expression levels and immune cell infiltration level

Description	EGFR		ERBB2		ERBB3		ERBB4	
	Cor	P	Cor	P	Cor	P	Cor	P
B-cell	0.1671	< 0.0001	0.02272	0.5527	−0.08882	0.0201	− 0.1591	< 0.0001
CD4-Tcell	−0.1143	0.0027	0.03041	0.4268	0.1200	0.0016	0.09663	0.0114
CD8-Tcell	0.05893	0.1233	0.07340	0.0549	−0.002242	0.9533	−0.01364	0.7217
Neutrophil	−0.04326	0.2582	0.06590	0.0848	−0.02445	0.5229	−0.06434	0.0925
Macrophage	0.07331	0.0551	0.1026	0.0072	−0.01798	0.6386	−0.05173	0.1763
Dendritic cell	−0.09997	0.0088	0.06210	0.1044	−0.01882	0.6229	−0.06135	0.1087

### PPI network of the EGFR family in glioma and enrichment analysis

**Fig. 7:**
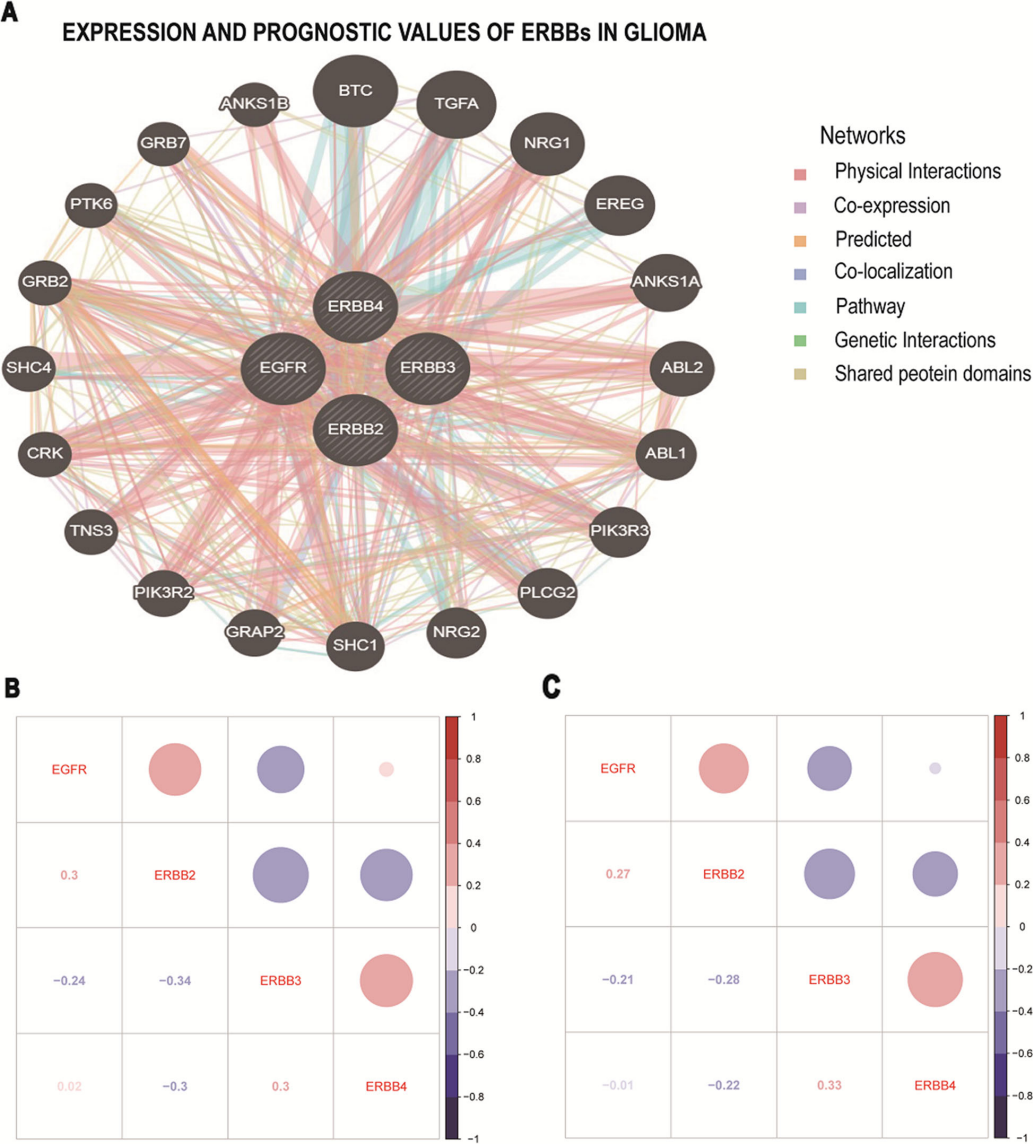
Predicted pathways and correlation of EGFR family gene expression in glioma. **a** A PPI network for the EGFR family was constructed in GeneMANIA. **b, c** Pearson’s test was used to study the correlations among the EGFR family members

Next, we used Gene-MANIA to construct a PPI network for the EGFR family, and the results are shown in Fig. 7a. Then, GO and KEGG analyses based on DAVID were performed to identify the functional enrichment of the EGFR family and their associated genes (Table 3). Biological process (BP) enrichment terms showed that the EGFR family and their interacting proteins were significantly associated with the ERBB2 signaling pathway, regulation of cell motility, regulation of phosphatidylinositol 3-kinase signaling, phosphatidylinositol phosphorylation, phosphatidylinositol-mediated signaling, peptidyl-tyrosine phosphorylation, epidermal growth factor receptor signaling pathway, MAPK cascade, transmembrane receptor protein tyrosine kinase signaling pathway, positive regulation of GTPase activity, wound healing, and positive regulation of cell proliferation. MF enrichment showed that the EGFR family was significantly correlated with phosphatidylinositol-4,5-bisphosphate 3-kinase activity, Ras guanyl-nucleotide exchange factor activity, epidermal growth factor receptor binding, ephrin receptor binding, and receptor signaling protein tyrosine kinase activity. KEGG enrichment revealed that the EGFR family was related to the ERBB signaling pathway, glioma, non-small cell lung cancer pathways, neurotrophin signaling pathways, chronic myeloid leukemia pathways, random signaling pathways in microRNAs in cancer tissues, and cancer lycans signaling pathways. Overall, the potential mechanisms by which the EGFR family participates in the carcinogenesis of glioma were explored by PPI construction and enrichment analysis.

**Table 3:** GO and KEGG enrichment analysis of the EGFR family and their 20 interactors

Category	Terms	Description	Count	FDR
BP	GO:0038128	ERBB2 signaling pathway	12	2.95E-21
BP	GO:2000145	regulation of cell motility	10	2.63E-17
BP	GO:0014066	regulation of phosphatidylinositol 3-kinase signaling	10	6.11E-13
BP	GO:0046854	phosphatidylinositol phosphorylation	10	3.83E-12
BP	GO:0048015	phosphatidylinositol-mediated signaling	10	1.18E-11
BP	GO:0018108	peptidyl-tyrosine phosphorylation	10	3.45E-10
BP	GO:0007173	epidermal growth factor receptor signaling pathway	8	1.01E-09
BP	GO:0000165	MAPK cascade	10	4.45E-08
BP	GO:0007169	transmembrane receptor protein tyrosine kinase signaling pathway	8	5.02E-08
BP	GO:0043547	positive regulation of GTPase activity	11	1.83E-06
BP	GO:0042060	wound healing	6	9.38E-05
BP	GO:0008284	positive regulation of cell proliferation	9	1.55E-04
MF	GO:0046934	phosphatidylinositol-4,5-bisphosphate 3-kinase activity	10	5.51E-14
MF	GO:0004713	protein tyrosine kinase activity	11	7.11E-13
MF	GO:0005088	Ras guanyl-nucleotide exchange factor activity	10	1.84E-11
MF	GO:0005154	epidermal growth factor receptor binding	6	5.20E-07
MF	GO:0046875	ephrin receptor binding	5	4.08E-05
MF	GO:0004716	receptor signaling protein tyrosine kinase activity	4	2.80E-04
KEGG	hsa04012	ERBB signaling pathway	18	2.00E-29
KEGG	hsa05214	Glioma	8	1.53E-07
KEGG	hsa05223	Non-small cell lung cancer	7	4.12E-06
KEGG	hsa04722	Neurotrophin signaling pathway	8	1.21E-05
KEGG	hsa05220	Chronic myeloid leukemia	7	1.93E-05
KEGG	hsa04014	Ras signaling pathway	9	4.27E-05
KEGG	hsa05206	MicroRNAs in cancer	9	2.66E-04
KEGG	hsa05205	Proteoglycans in cancer	8	4.15E-04
KEGG	hsa04510	Focal adhesion	8	5.07E-04
KEGG	hsa05215	Prostate cancer	6	0.002482
KEGG	hsa05200	Pathways in cancer	9	0.003013
KEGG	hsa04915	Estrogen signaling pathway	6	0.004453
KEGG	hsa05213	Endometrial cancer	5	0.00888

BP, Biological processes; MF, molecular functions; KEGG, Kyoto Encyclopedia of Genes and Genomes; GO, gene ontology; FDR, false discovery rates

### The relationship between EGFR family alterations and prognosis in patients with glioma

To further understand the EGFR family, we used the cBioPortal online tool to study the alterations of the EGFR family and their correlation with prognosis. We found that among 885 patients with glioma, 272 patients had alterations in EGFR family genes (31%), and the most common genetic alteration was amplification (Fig. 1a). In addition, the cBioPortal database showed the correlations between EGFR family genetic alterations and overall survival (OS) (*p* < 0.001), disease-free survival (DFS) (p < 0.001), disease-specific survival (DSS) (p < 0.001), and progression-free survival (PFS) of patients with glioma (p < 0.001) (Fig. 1b, c, d, and e). We also used Pearson’s test to study the correlations among the expression levels of the EGFR family in the CGGA and TCGA datasets. We found some EGFR family members to be notably positively correlated: EGFR with ERBB2; ERBB3 with ERBB4. Some other EGFR family members were significantly negatively correlated: EGFR with ERBB3; ERBB2 with ERBB3 and ERBB4. The expression of EGFR had no association with ERBB4 (Fig. 7b and c).

### Prognostic values of the EGFR family in glioma

**Fig. 8:**
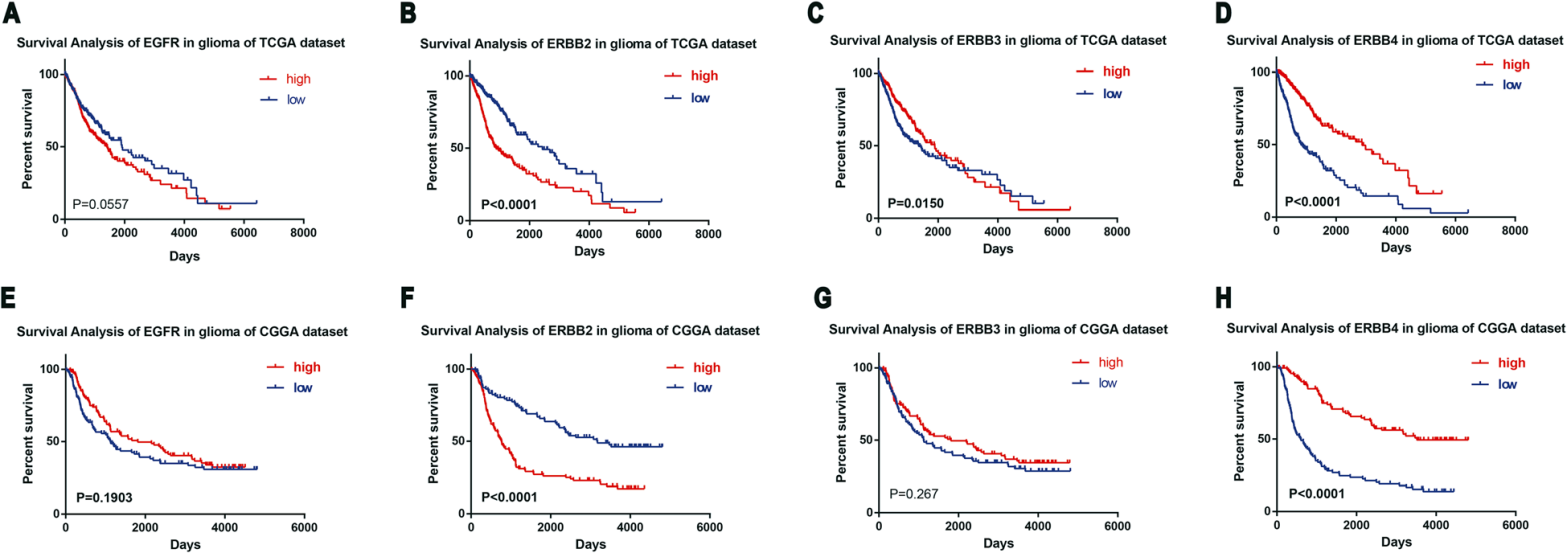
The prognostic value of the EGFR family in glioma. **a, b, c** and **d** Overall survival correlated with the expression of the EGFR family members in all grades of glioma in the TCGA dataset. **e, f, g** and **h** Overall survival correlated with the expression of EGFR family members in all grades of glioma in the CGGA dataset

In addition, we used the TCGA and CGGA datasets to evaluate the prognostic impact of EGFR family expression on high-grade glioma. The results showed that high mRNA levels of ERBB2 and ERBB4 in glioma patients were associated with a poor prognosis (Fig. 8c, d, g and h), while the expression of EGFR and ERBB3 had no correlation with the prognosis of glioma patients (Fig. 8a, b, e and f). The results from the CGGA and TCGA data sets were similar. Next, through univariate and multivariate Cox analysis, we also found that age, tumor grade, IDH mutations and EGFR family member expression levels were prognostic factors of glioma patients (Table 4).

**Table 4:** Univariate and multivariate regression analysis was used to predict the overall survival rate of glioma patients

Characteristic	TCGA (*n* = 668)			CGGA (*n* = 326)		
	P	HR	95%CI	P	HR	95%CI
Univariate						
Age	< 0.0001	1.068	1.057–1.078	< 0.0001	1.054	1.038–1.070
Gender	0.144	0.826	0.639–1.068	0.806	1.044	0.741–1.469
Grade	< 0.0001	4.638	3.803–5.657	< 0.0001	1.952	1.708–2.232
IDH	< 0.0001	0.127	0.096–0.169	< 0.0001	2.802	1.960–4.007
EGFR	< 0.0001	1.187	1.103–1.278	0.808	1.013	0.914–1.123
ERBB2	< 0.0001	2.177	1.884–2.516	< 0.0001	1.961	1.613–2.384
ERBB3	0.001	0.879	0.818–0.946	0.005	0.866	0.783–0.958
ERBB4	< 0.0001	0.684	0.638–0.734	< 0.0001	0.630	0.564–0.703
Multivariate						
Age	< 0.0001	1.033	1.021–1.044	0.001	1.029	1.012–1.046
Gender	0.596	0.931	0.715–1.212	0.482	0.882	0.620–1.253
Grade	< 0.0001	2.218	1.727–2.848	< 0.0001	1.752	1.485–2.066
IDH	< 0.0001	0.341	0.235–0.495	0.429	1.192	0.771–1.841
EGFR	0.459	0.978	0.921–1.038	0.776	1.012	0.931–1.100
Age	< 0.0001	1.032	1.020–1.044	0.002	1.027	1.010–1.044
Gender	0.537	0.920	0.706–1.199	0.531	0.894	0.629–2.067
Grade	< 0.0001	2.185	1.700–2.809	< 0.0001	1.751	1.483–2.067
IDH	< 0.0001	0.375	0.253–0.554	0.876	1.036	0.662–1.622
EGFR2	0.142	1.128	0.960–1.326	< 0.0001	1.464	1.199–1.786
Age	< 0.0001	1.034	1.022–1.045	0.001	1.029	1.012–1.045
Gender	0.646	0.940	0.722–1.224	0.491	0.884	0.621–1.257
Grade	< 0.0001	2.288	1.780–2.942	< 0.0001	1.745	1.479–2.059
IDH	< 0.0001	0.335	0.231–0.484	0.437	1.188	0.768–1.835
EGFR3	0.032	1.077	1.006–1.153	0.766	0.986	0.896–1.084
Age	< 0.0001	1.033	1.021–1.045	0.001	1.027	1.011–1.043
Gender	0.623	0.936	0.719–1.218	0.678	0.927	0.650–1.323
Grade	< 0.0001	2.282	1.755–2.967	< 0.0001	1.575	1.322–1.876
IDH	< 0.0001	0.333	0.227–0.488	0.329	1.248	0.800–1.946
EGFR4	0.396	1.041	0.949–1.141	< 0.0001	0.757	0.664–0.864

## Discussion

Glioma is an invasive and highly diffuse brain tumor [38]. Current standard treatment for glioma patients includes maximum safe surgical resection, simultaneous radiotherapy and temozolomide, and then adjuvant temozolomide. Glioma is still an incurable disease; the average OS after standard treatment is 12–15 months, and relapse is inevitable [39]. Therefore, it is extremely important to explore new methods to improve the quality of life and survival times of glioma patients.

Research results in recent years have shown that the tumor microenvironment plays an important role in the occurrence and development of glioma. An in-depth understanding of the tumor microenvironment is beneficial to provide new immunotherapy for glioma patients to inhibit tumor development [40, 41]. In recent years, immune checkpoint inhibitors against members of the EGFR family have been widely tested against gliomas in clinical trials, opening up broad new prospects for the treatment of gliomas [42, 43]. In this study, we analyzed the expression of EGFR family members in gliomas and their relationships with prognosis and immune infiltration. Our results suggest that the EGFR family mRNA expression levels are related to the poor prognosis of glioma. In addition, EGFR family mRNA levels are correlated with the abundance of tumor-infiltrating immune cells. Overall, our study provides new insights into the important roles of the EGFR family members in the assessment of glioma prognosis and immune infiltration.

The abnormal expression of the EGFR family in a significant proportion of human cancers has been studied; however, the roles of the EGFR family members in gliomas is still uncertain [44, 45]. Here, to clarify the expression profile of the EGFR family members in all grades of gliomas, by analyzing glioma samples in the CGGA and TCGA datasets, we summarized the expression patterns and distribution of the EGFR family. We found that the expression of the EGFR family in glioma had significant changes at the mRNA levels. At the same time, the expression patterns of the EGFR family members in various subtypes of gliomas were significantly different, suggesting that the EGFR family is related to the malignant phenotype and tumor progression. In addition, the EGFR family member expression pattern is significantly different in IDH-mutated gliomas, suggesting that IDH may be a regulator of the EGFR family.

After binding and activation, EGFR can form a dimer structure with other members. They all preferentially bind to ERBB2 to form a stronger heterodimer. This initiates a series of cascade reactions through autophosphorylation, which participate in cell signal transmission. These signals reach the nucleus and they play important roles in normal cell proliferation, differentiation and migration [46]. EGFR gene amplification and overexpression can be seen in a variety of human malignancies, including non-small cell lung cancer [47], breast cancer [48], ovarian cancer [49], gastric cancer [50], etc. Abnormal EGFR gene activation is closely related to tumor cell proliferation, angiogenesis, tumor invasion and migration, and inhibition of apoptosis [51].

ERBB2 forms a heterodimer with other members of the family. This allows it to bind indirectly to the ligand, which activates the tyrosine kinase in its intracellular segment, triggering downstream signal transduction. The signal is transmitted to the nucleus through intercellular substances, activating cell proliferation-related genes, thereby promoting cell mitosis and modulating cell proliferation, differentiation, migration and tumor formation [52, 53]. ERBB2 is overexpressed to varying degrees in many malignancies, such as breast cancer [54], ovarian cancer [55], non-small cell lung cancer [56], and gastric cancer [57]. Besides, ERBB2 is upregulated in high grade gliomas and correlated with PD-L1 expression [58].

The ERBB3/ERBB2 dimer is the most active ERBB dimer, and it can activate the PI3K/AKT, Jak/Stat and other signaling pathways and regulate cell proliferation, differentiation, migration and other activities [59]. ERBB3 is closely related to the occurrence and development of various tumors. Abnormal activation and overexpression of the HER3 gene can be seen in malignant tumors such as breast cancer [60], gastric cancer [61], ovarian cancer [62], and prostate cancer [63].

After ligands bind to ERBB4 (neurodifferentiation factor heparin binding epidermal growth factor, etc.), it activates downstream PI3K/Akt and Ras/Raf/MAPK signaling pathways through autophosphorylation and mediates extracellular growth factor signaling through intracellular kinase cascade intracellular transmission, thereby regulating angiogenesis and cell growth, differentiation, proliferation and apoptosis [64].

In recent years, the importance of immune cell infiltration in tumors has gradually been recognized [65, 66]. Blocking immune checkpoints has become a promising cancer treatment [67]. However, the relationship between the EGFR family and immune infiltration in gliomas has not been studied. In this paper, the TIMER database was used to analyze the relationship between EGFR family expression levels and immune penetration in gliomas. The expression of EGFR has a notable correlation with the level of B cell infiltration. ERBB2 expression was notably correlated with the level of macrophage infiltration. The expression of ERBB3 and ERBB4 were positively correlated with the level of CD4+ T cell infiltration. These correlations may suggest a potential mechanism by which the EGFR family regulates glioma immune cells. These findings indicate that the EGFR family plays a crucial role in the regulation of glioma immune cells.

To explore the potential mechanism of EGFR family involvement in glioma carcinogenesis, we constructed a PPI network and performed GO and KEGG analyses of the EGFR family with DAVID. The results showed that EGFR family interacting genes are mainly involved in cell motility, which may affect integrin receptors and Rho family GTPases. Integrin receptors have been reported to interact with EGFR [68]. Moreover, Rho family GTPases play an important role in the interactions between the EGFR family members and other proteins. In summary, the interaction between integrin receptors/Rho family GTPases and the EGFR family may become a new antitumor therapy strategy that can regulate signaling pathways [69].

## Conclusion

This study systematically analyzed the expression patterns of the EGFR family, their mutations, and their correlations with the prognosis of patients with glioma, advancing our understanding of the biological characteristics of glioma. These results revealed that the EGFR family might play an important role in the development of gliomas. The EGFR family members can also be used as molecular markers for glioblastoma, may be potential biomarkers for the diagnosis and prognosis of patients with glioma, and may be therapeutic targets for the treatment of glioma.

## Data Availability

The datasets analyzed for this study were obtained from the Oncomine database (https://www.oncomine.org/), the UCSC Xena website (https://xenabrowser.net/datapages/) and the CGGA dataset (mRNAseq_325) (http://www.cgga.org.cn/index.jsp), the TIMER (https://cistrome.shinyapps.io/timer/) website, the cBioPortal (https://www.cbioportal.org/) and the GeneMANIA databases (http://www.genemania.org/), and DAVID (https://david.ncifcrf.gov/).

## Abbreviations

EGFR: Epidermal growth factor receptor
GBM: Glioblastoma
CNS: Brain and central nervous system
PPI: Protein-protein interaction
MF: Molecular function
CC: Cell composition
BP: Biological process
OS: Overall survival
DFS: Disease-free survival
DSS: Disease-specific survival
PFS: Progression-free survival
